# A molecular dynamics study of the oxidation mechanism, nanostructure evolution, and friction characteristics of ultrathin amorphous carbon films in vacuum and oxygen atmosphere

**DOI:** 10.1038/s41598-021-81659-w

**Published:** 2021-02-16

**Authors:** Shengxi Wang, Kyriakos Komvopoulos

**Affiliations:** Department of Mechanical Engineering, University of California, Berkeley, CA 94720 USA

**Keywords:** Engineering, Materials science, Nanoscience and technology, Optics and photonics

## Abstract

Amorphous carbon (*a*-C) films are characterized by extraordinary chemical inertness and unique thermophysical properties that are critical to applications requiring oxidation-resistant, low-friction, and durable overcoats. However, the increasing demands for ultrathin (a few nanometers thick) *a*-C films in various emerging technologies, such as computer storage devices, microelectronics, microdynamic systems, and photonics, make experimental evaluation of the structural stability and tribomechanical properties at the atomic level cumbersome and expensive. Consequently, the central objective of this study was to develop comprehensive MD models that can provide insight into the oxidation behavior and friction characteristics of ultrathin *a*-C films exhibiting layered through-thickness structure. MD simulations were performed for *a*-C films characterized by relatively low and high *sp*^3^ contents subjected to energetic oxygen atom bombardment or undergoing normal and sliding contact against each other in vacuum and oxygen atmosphere. The effect of energetic oxygen atoms on the oxidation behavior of *a*-C films, the dependence of contact deformation and surface attractive forces (adhesion) on surface interference, and the evolution of friction and structural changes (rehybridization) in the former *a*-C films during sliding are interpreted in the context of simulations performed in vacuum and oxidizing environments. The present study provides insight into the oxidation mechanism and friction behavior of ultrathin *a*-C films and introduces a computational framework for performing oxidation/tribo-oxidation MD simulations that can guide experimental investigations.

## Introduction

The unique properties and microstructure of amorphous carbon (*a*-C) films have led to their usage in many applications requiring protective overcoats and thin-film structures that demonstrate high strength, optical transparency in the visible and near-infrared wavelength range, thermal stability, chemical inertness, and biocompatibility^1–6^. In principle, carbon atom hybridization is characterized by three different atomic bonding configurations (*sp*^3^, *sp*^2^, and *sp*^1^), with most *a*-C films exhibiting predominantly either graphite-like (*sp*^2^) or diamond-like (*sp*^3^) carbon microstructures^7^, depending on the deposition conditions. The amorphous structure and most properties of *a*-C films are closely correlated with the fraction of *sp*^3^ bonding, which can be tailored by adjusting the film growth conditions and doping with various elements, such as hydrogen, nitrogen, and metallic elements^8–11^. The superior tribomechanical properties (e.g., low friction and high hardness) of *a*-C films with significant *sp*^3^ bonding fractions have led to their extensive usage in a wide range of applications where preserving the substrate integrity and minimizing friction at contact interfaces are of critical importance to the reliability and operation efficiency of contact-mode electromechanical devices and mechanical components^12,13^.

The performance of *a*-C films under various ambient conditions is another critical factor of their effectiveness as protective overcoats in harsh environments, where maintaining the thermal stability and chemical inertness of thin-film structures is challenging. The environmental response of *a*-C films can be generally characterized by changes in the morphology, microstructure, and properties, depending on the temperature, atmosphere, and type of dopant(s). For instance, the decrease of the optical gap and the resistivity of *a*-C films as a result of thermal annealing has been correlated with the mobility of *sp*^2^ sites and the growth of larger aromatic clusters^14^.

The increasing interest to apply *a*-C films as protective, low-friction overcoats in many pioneering technologies has concentrated the attention of researchers on the structural stability, oxidation resistance, and tribological properties of *a*-C films under different environmental conditions, particularly elevated temperatures. Characteristic application examples where the thermal stability of *a*-C films is of critical importance include optical data storage, heat-assisted magnetic recording, and low earth orbit spaceships, where operation at a high temperature, oxidation due to laser heating, and high-speed particle collision, respectively, can be detrimental to the endurance and functionality of the protective film^15–17^. An elevated temperature may destabilize the microstructure and degrade the properties of *a*-C films by promoting *sp*^3^-to-*sp*^2^ rehybridization, consequently lessening the film’s ability to effectively protect the substrate material^18,19^. Experimental and computational studies have revealed the existence of a threshold temperature in the range of 200–350 °C above which *a*-C films undergo significant structural changes^20–22^. Increasing the *sp*^3^ fraction and doping with certain elements, such as silicon, have been proven effective methods for enhancing the thermal stability of *a*-C films by stabilizing the carbon atoms in the *sp*^3^ hybridization state and inhibiting *sp*^3^-to-*sp*^2^ rehybridization at high temperatures^23–25^. The significant effect of environmental conditions (e.g., humidity) on the tribological properties of *a*-C films^26,27^ prompted experimental studies in vacuum and various atmospheres, including ambient and dry air, or pure gas, such as hydrogen, nitrogen, and carbon dioxide^28,29^. Macroscopically, *a*-C films exhibit lower friction in ambient air than dry air and even lower friction in hydrogen and carbon dioxide atmospheres, but significantly higher friction in vacuum and nitrogen environments. The low friction of *a*-C films has been attributed to surface passivation by gaseous species or functional groups from the atmosphere^30,31^. The termination of the free dangling bonds at the film surface by dissociated gaseous species or functional groups may significantly reduce the adhesion force at contact interfaces. These findings have shown that even though adsorption and dissociation of hydrogen, oxygen, and water molecules can readily occur at the surface of *a*-C films, nitrogen dissociation is not a preferential process.

Although the foregoing studies have provided valuable insight into the behavior of *a*-C films under various environmental conditions, the multiple types of carbon bonding configurations and the vastly diverse operation conditions make thorough investigation of the underlying physicochemical processes expensive and often impossible. Therefore, insight into the structural changes and tribological behavior of ultrathin films exposed to different temperatures and environments requires atomic-scale approaches. The problem is further perplexed by the layered structure of *a*-C films grown by deposition methods that employ energetic particle bombardment^22^. In view of the challenges associated with structural characterization and the quantification of the properties of films at the atomic level, atomistic computational methods have been developed to bridge the gap in fundamental knowledge about the effect of atmospheric conditions on the structural stability and tribological behavior of ultrathin films. Among various computational methods, molecular dynamics (MD) has been proven to be a particularly effective method for studying spatiotemporal structural changes in material systems at atomic length scales and femtosecond time scales. The supremacy of MD analysis over most experimental methods has enabled the procurement of basic insight into the environmental behavior of ultrathin *a*-C films. For example, MD simulations have shown that friction of *a*-C and *a*-C:H films is controlled by chemical reactions instigated between unsaturated carbon atoms in the film structure adjacent to the contact interface^32^. The mechanisms responsible for lowering friction via surface passivation by hydrogen, fluorine, carbon dioxide, nitrogen, and water have been studied by MD and quantum mechanics. The direct observation of bond formation and breakage and the calculation of interatomic energies in these studies have indicated that surface passivation is a consequence of stress-induced activation and dissociation of the adsorbed molecules^28,29,33^. As an example, the adsorption energies and configurations of various gases on graphene were investigated by MD simulations and the stress developed into the graphene substrate due to the adsorption/desorption of these species was determined from a simple theoretical method^34^. MD simulations of the tribochemical behavior of *a*-C films at the mesoscale have revealed tribo-emission of trace amounts of hydrocarbon molecules due to asperity contact interaction^35,36^, which is difficult to track experimentally. The insight into the tribochemical characteristics of *a*-C films provided by the foregoing investigations and several others indicate that MD offers unique capabilities for examining structural changes and tribochemical phenomena at the atomic level, which is the appropriate level for studying the behavior of films that are a few nanometers thick and fast occurring processes.

Despite the abundance of studies dealing with the structural stability and tribological behavior of *a*-C films in various environments, inclusive studies of the oxidation and tribo-oxidation processes encountered with ultrathin *a*-C films are sparse, even though harsh environmental conditions are typical in several leading-edge technologies. Therefore, the objective of this study was to perform comprehensive MD simulations of the oxidation and tribo-induced oxidation processes and the friction behavior of < 2-nm-thick *a*-C films exposed to elevated temperature and oxidizing atmosphere. The MD simulations of this study comprised two phases. First, oxidation of ultrathin *a*-C films with low or high *sp*^3^ contents deposited on a diamond substrate was simulated to investigate the effect of oxygen kinetic energy on the oxidation resistance of different film nanostructures. Second, normal and sliding contact of similar ultrathin *a*-C films with or without adsorbed oxygen was simulated for a range of surface interference (normal force) to elucidate the friction behavior and the effect of the sliding process on tribo-induced oxidation of the ultrathin *a*-C films. The simulation results presented below establish a computational framework that can be useful in guiding the development of oxidation-resistant, low-friction films used as protective overcoats in high-temperature and oxidizing environments.

## Computational procedure

### Oxidation model

A 15.13 × 15.13 × 92 Å simulation box with periodic *x-* and *y*-boundary conditions was used in the oxidation simulations. The first phase of these simulations comprised the growth of ultrathin *a*-C films on a 15.13 × 15.13 × 8 Å diamond (100) substrate, simulated by the sequential deposition of single carbon atoms of various kinetic energies. Energetic carbon atom implantation resulted in crystalline-to-amorphous transformation of the top 5 Å of the diamond substrate. The subsequent carbon atom impingement onto the amorphized diamond surface led to the growth of an *a*-C film. Thus, the MD model encompassed a three-layer architecture consisting of crystalline diamond substrate, amorphous diamond (intermixing) layer, and ultrathin *a*-C film. For simplicity, the amorphous diamond layer and the *a*-C film will be hereafter referred to as the amorphous carbon structure. The overall *sp*^3^ content of the *a*-C film was varied by changing the kinetic energy of the incident carbon atoms. Additional details about the MD model of ultrathin *a*-C film growth used in this study can be found elsewhere^22^.

**Figure 1:**
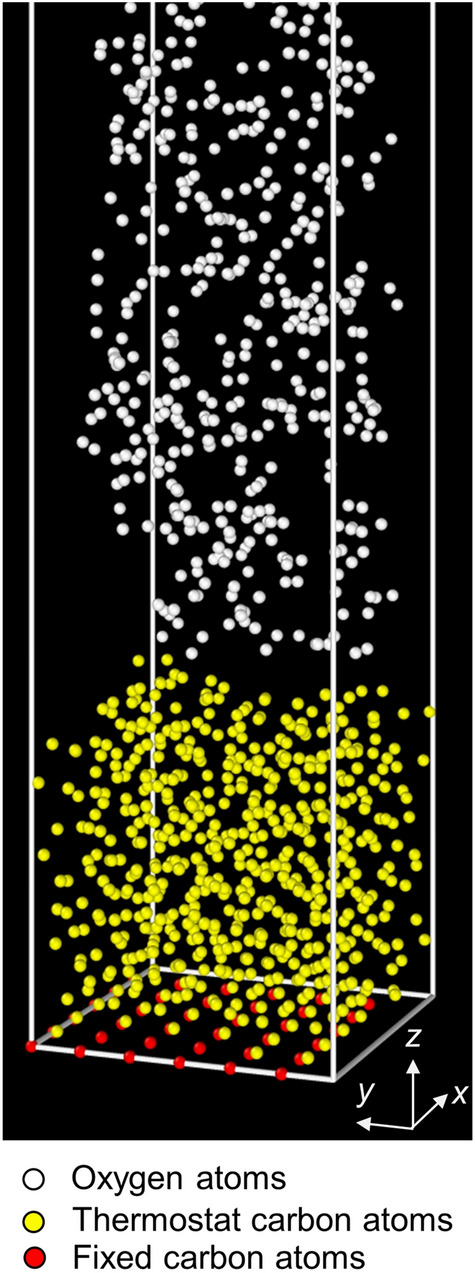
MD model of *a*-C film oxidation consisting of *a*-C film (red and yellow atoms) and 500 oxygen (white) molecules. The red carbon atoms at the bottom of the film were fixed to simulate a semi-infinite diamond bulk substrate. The yellow atoms were not constrained but were assigned to a Berendsen thermostat to control the temperature. The kinetic energy of the oxygen molecules was controlled by a second Berendsen thermostat.

In the second phase of the oxidation simulations, 500 oxygen molecules were randomly generated in the space above the *a*-C film, as shown in Fig. 1. The bottom atoms of the diamond substrate were fixed to simulate a semi-infinite medium, whereas the remaining carbon atoms were allowed to move freely; however, they were also assigned to a Berendsen thermostat with a temperature damping constant of 10 fs to maintain the temperature in the film at 573 K during the simulation. The total number of particles in this MD model is ∼1800, of which − 800 particles are carbon atoms. The oxidation process was simulated for 1000 ps using a time step of 0.2 fs. While the number of carbon atoms involved in the oxidation process must be controlled to avoid excessive computation, the number of oxygen molecules must be sufficiently high to maintain a continuous carbon–oxygen interaction process and a realistic computation time. The kinetic energy of the oxygen molecules was varied in the range of 1–20 eV.

An empirical reactive force-field method, termed ReaxFF^37^, which allows for fully reactive atomistic scale MD simulations that encompass chemical reactions, was used in the present study. It has been argued that the ReaxFF potential can be used to describe Si, SiO_2_, diamond, graphite/graphene, and a wide range of Si/C/O/H material systems^38^. The input parameters of the ReaxFF interatomic potential are usually obtained by fitting a training data set of both quantum mechanics and experimental data. The combination of energy accuracy and low computation cost makes the ReaxFF method suitable for chemically reactive systems. The input parameters of the ReaxFF potential used in this study were quoted from the literature^38^.

### Tribo-oxidation model

**Figure 2:**
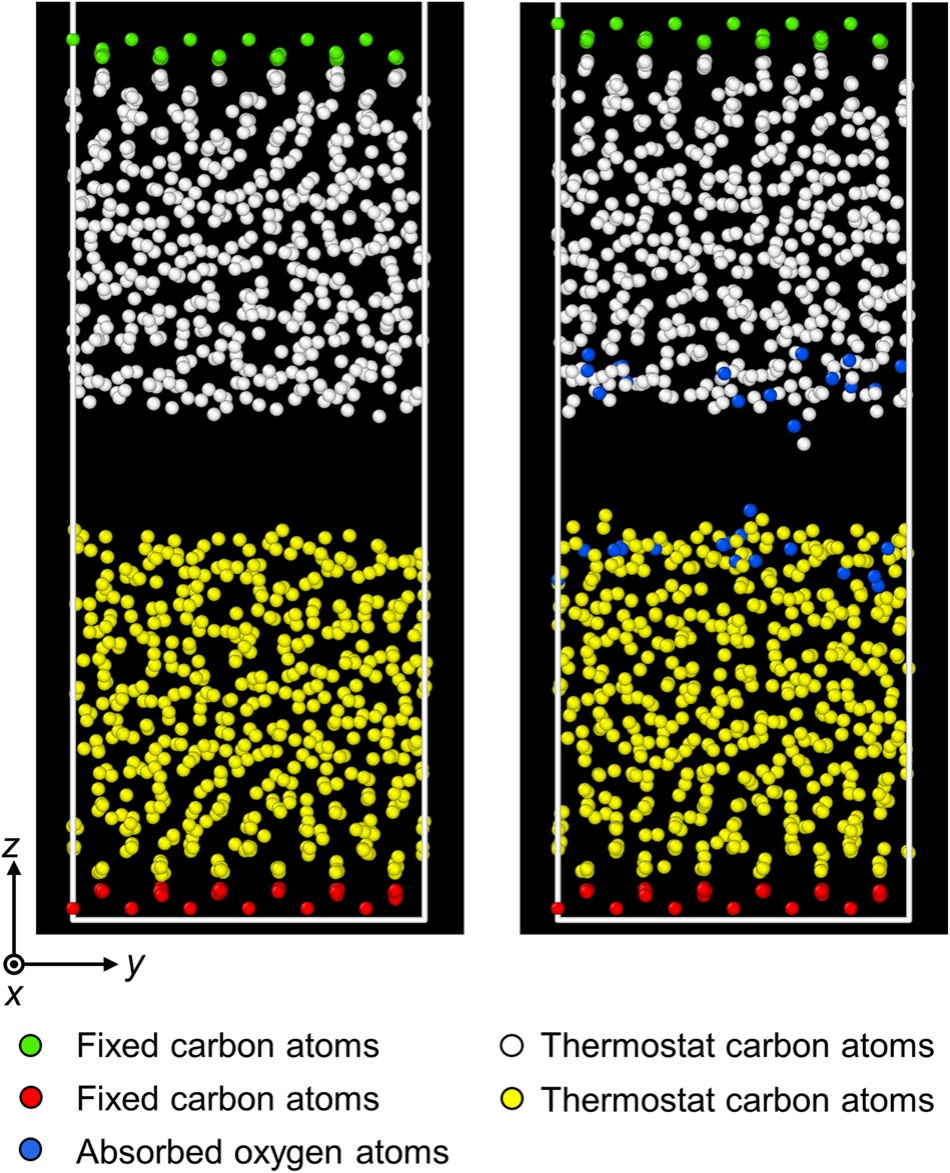
MD sliding models of two identical *a*-C films interacting in vacuum (left) and oxygen atmosphere (right). The green and red atoms at the bottom of the upper and lower films, respectively, were fixed to simulate semi-infinite diamond substrates. The white and yellow atoms of the films were not constrained, but assigned to Berendsen thermostats to control the temperature during the simulation. The blue atoms (right) are oxygen atoms adsorbed into the film surfaces during sliding in the oxygen atmosphere. The simulations comprised the sequential phases of normal and sliding contact, involving the downward movement of the upper film in the *z*-direction and subsequent sliding in the *y*-direction, while the bottom film was kept stationary.

To investigate the normal and sliding contact behaviors of ultrathin *a*-C films in vacuum and oxygen atmosphere, a 15.13 × 15.13 × 65 Å simulation box with periodic *x*- and *y*-boundary conditions was used to simulate sliding between two similar *a*-C films (Fig. 2), which were grown in the same way as the *a*-C film described in the previous section. Similarly with the oxidation model, the bottom atoms of the lower film and the top atoms of the upper film were fully constrained to simulate semi-infinite media, whereas the remaining atoms were left unconstrained, but assigned to Berendsen thermostats with a temperature damping constant equal to 20 fs to maintain the temperature in the films at 573 K. Initially, the two films were placed apart by a distance of 5 Å. In the simulations of normal contact in vacuum, the upper film was displaced towards the stationary lower film at a constant speed of 40 m/s up to a maximum surface interference of 6.4 Å. After allowing the system to equilibrate for 10 ps at the set maximum interference, the upper film was retracted at the same constant speed until it fully separated from the lower film. In the simulations of sliding contact in vacuum, the upper film was again displaced towards the lower film at a constant speed of 40 m/s to a maximum surface interference in the range of 1–6 Å and after equilibrating for 10 ps at the specified interference, it was traversed laterally at a constant speed of 20 m/s, while keeping the interference fixed. After sliding for a total distance of 40 Å, i.e., more than two times the lateral dimension of the simulation box, the two films were fully separated by retracting the upper film at the same speed as for the loading. To simulate normal and sliding contact in an oxidizing environment, the upper and lower *a*-C films were initially kept in an oxygen atmosphere of 573 K that contained enough oxygen molecules until oxygen adsorption reached equilibrium. Subsequently, the excess free oxygen molecules were removed from the simulation box and normal or sliding contact was simulated using the same parameters as in the vacuum simulations.

In the foregoing simulations, carbon–carbon interaction was described by the Tersoff potential, whose parametrization is detailed elsewhere^39^ and its ability to simulate *a*-C systems has been previously verified^22,40^. The carbon–oxygen and oxygen–oxygen interactions were described by the Lennard–Jones potential with parameters given elsewhere^41,42^. All of the MD simulations were performed with the Large-scale Atomic/Molecular Massively Parallel Simulator (LAMMPS) software.

## Results and discussion

### Oxidation mechanism

**Figure 3:**
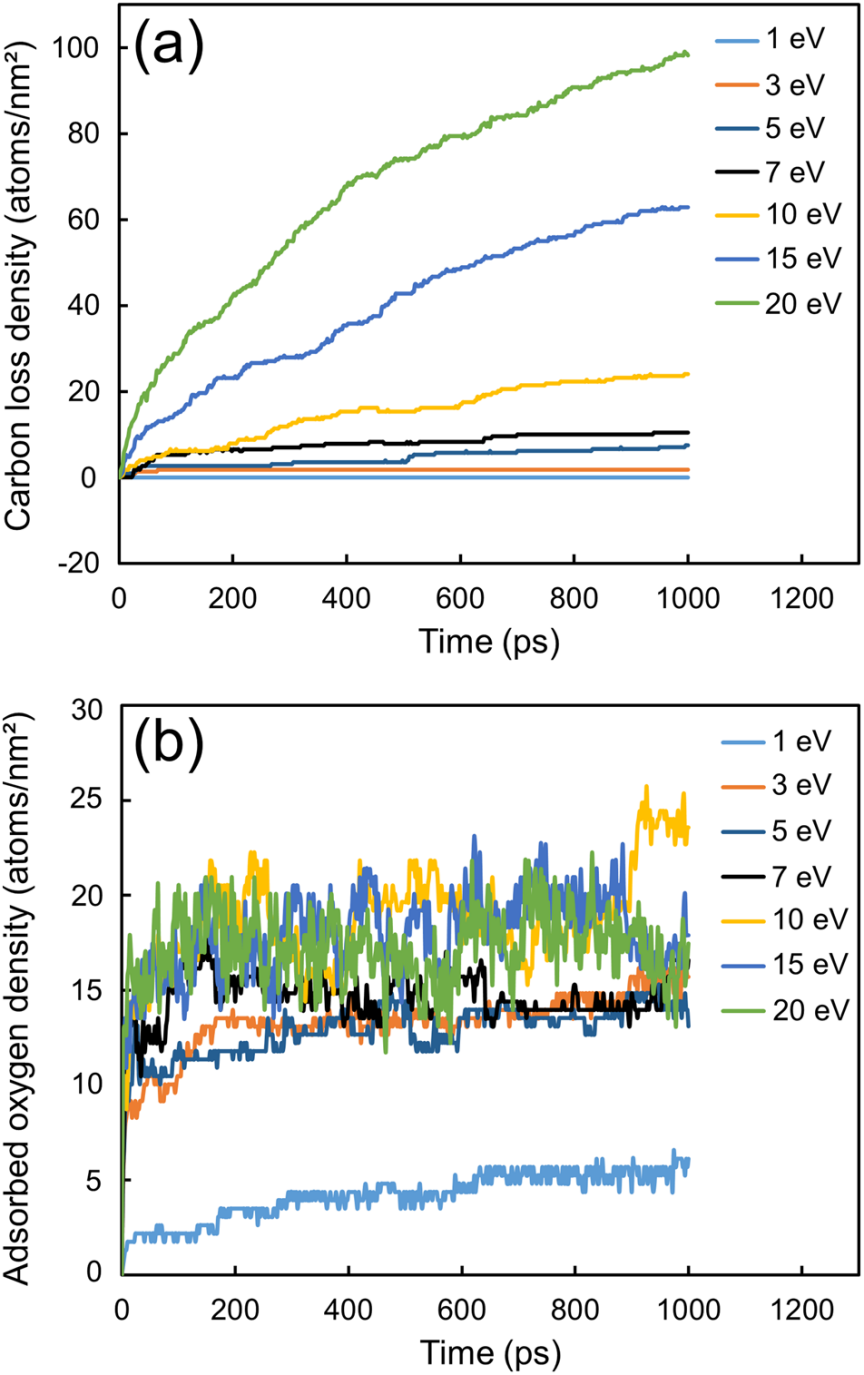
(**a**) Carbon loss density and (**b**) oxygen adsorption density versus oxidation time for *a*-C film with 66% *sp*^3^ content and oxygen kinetic energy in the range of 1–20 eV. The carbon atoms were removed from the *a*-C film as CO and CO_2_ gaseous products. The initial surface area density of the *a*-C film is 270 atoms/nm^2^.

Figure 3 shows the carbon loss density and the adsorbed oxygen density versus the oxygen kinetic energy for an *a*-C film with 66% *sp*^3^ content. The carbon loss represents the carbon atoms removed from the film in the form of gaseous products, i.e., CO or CO_2_, and may be inferred as being indicative of the depletion of the structural integrity and protection performance of the film. While both carbon loss and oxygen adsorption were instigated with the onset of oxidation, they demonstrated distinctly different dependencies on oxidation time and oxygen kinetic energy. As shown in Fig. 3a, the carbon loss density increased monotonically with time, showing a strong dependence on oxygen kinetic energy. Specifically, for an oxygen kinetic energy of ≤ 7 eV, the carbon loss at the end of the simulation was less than 4% of the total carbon atoms comprising the film. In fact, the carbon loss for 1 and 3 eV was nearly zero throughout the oxidation process. For oxygen kinetic energies above 7 eV, however, the carbon loss at the end of the simulation increased proportionally with the oxygen kinetic energy; particularly, for oxygen kinetic energy equal to 10, 15, and 20 eV, the carbon atom loss was 8.0%, 20.9%, and 32.7% of the total number of carbon atoms in the film, respectively. This reveals a critical kinetic energy of ∼7 eV for continuous carbon loss due to oxidation. Figure 3b shows that the adsorbed oxygen density reached rapidly a level where it exhibited fluctuations that intensified with the increase of the oxygen kinetic energy. A remarkable increase of the oxygen adsorption density occurred as a result of the increase of the oxygen kinetic energy from 1 to 3 eV, revealing the existence of a critical oxygen kinetic energy for significant oxygen adsorption. Indeed, all of the steady-state oxygen adsorption densities exhibited fluctuations in the range of 10–20 atoms/nm^2^, except for 1 eV oxygen kinetic energy for which the steady-state oxygen adsorption density varied slightly at the 5 atoms/nm^2^ level, implying surface saturation by adsorbing oxygen molecules independent of oxygen kinetic energy above ~ 1 eV. Importantly, oxygen surface adsorption was found to be dissociative in all simulation cases, suggesting that the oxygen bonds dissociated upon the adsorption of the oxygen molecules at the film surface. Hence, the rapid saturation of oxygen adsorption is attributed to the fixed density of dangling bonds at the film surface for oxygen atoms to attach and the dynamic equilibrium of oxygen adsorption at the simulated film temperature. Since 1 eV is much less than the dissociation energy of the oxygen bond (5.15 eV), it may be inferred that the oxygen adsorption density at 1 eV was less than those at oxygen kinetic energy above 5 eV.

**Figure 4:**
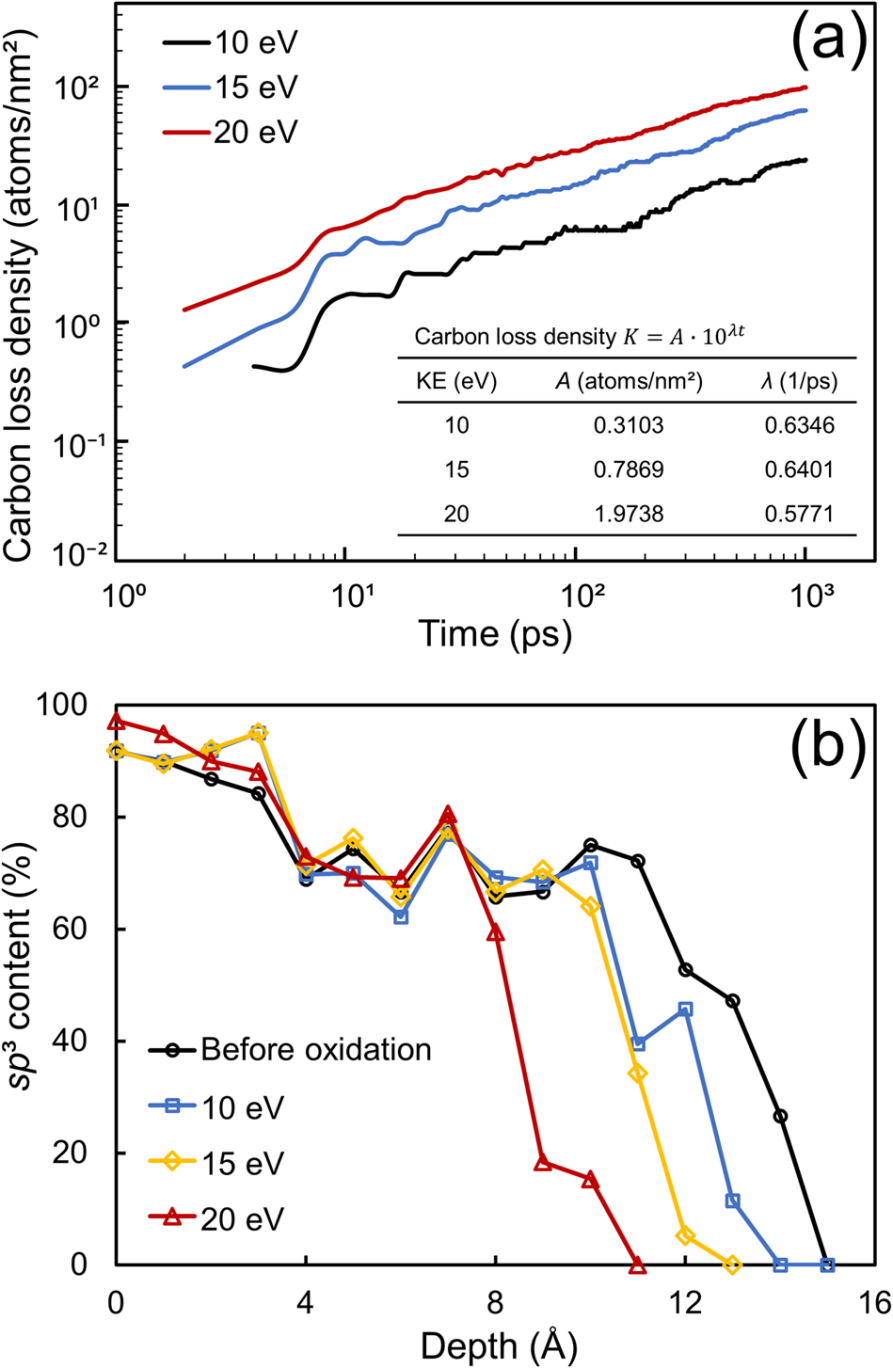
(**a**) Carbon loss density versus oxidation time and (**b**) depth profiles of the *sp*^3^ content of *a*-C film with 66% *sp*^3^ content obtained before and after 400 ps of oxidation for an oxygen kinetic energy equal to 10, 15, and 20 eV. The zero depth position in (**b**) corresponds to the bottom atomic plane of the film (the fixed red atoms in Fig. 1).

To examine the kinetics of the oxidation process, the carbon loss density versus time was graphed on a log–log plot, as shown in Fig. 4a for oxygen kinetic energy equal to 10, 15, and 20 eV. The parameters obtained from exponential fits through the data of each simulation are given in the inset of the figure. The plotted curves show that the coefficient *A* increased with the oxygen kinetic energy, whereas the exponent *λ* remained fairly constant. Figure 4b shows *sp*^3^ depth profiles for an *a*-C film with 66% *sp*^3^ content obtained before and after oxidation for 400 ps at various oxygen kinetic energies. The profiles were computed by averaging the *sp*^3^ fraction of 1-Å-thick slices through the film thickness. The *sp*^3^ depth profile before oxidation reveals a layered structure consisting of diamond substrate, bulk layer, and surface layer, characterized by nearly 100% *sp*^3^, roughly constant and relatively high *sp*^3^, and sharply decreasing *sp*^3^, respectively, confirming the formation of a layered structure in the film-growth simulation phase, in agreement with experimental findings of a layered *a*-C film structure produced by deposition methods that use energetic particles as film precursors^43^. Notably, in spite of the decrease of the film thickness due to the significant loss of carbon after oxidation for 400 ps, the layered film structure was still preserved. The high *sp*^3^ content of the bulk layer is a consequence of a highly compressive mechanical environment instigated by the intense carbon atom bombardment during film growth and the spatial constraint imposed by above-deposited atoms, whereas the low *sp*^3^ content of the surface layer is due to the development of a tensile stress, a consequence of considerably less atomic bombardment compared to the bulk layer and the relaxation of the film surface instigated by the reduced spatial constraint of near-surface carbon atoms^40^. During the oxidation process, the energetic oxygen molecules dissociating at the film surface upon adsorption continuously consumed the carbon atoms of the surface layer, causing the surface layer to become thinner. Since the latter weakened the spatial constraint on the bulk layer, it relaxed its top region, activating a phase transformation process characterized by *sp*^3^-to-*sp*^2^ rehybridization, which converted the top region of the bulk layer to a new surface layer. Because this process maintained a fairly constant thickness of the surface layer, the high *sp*^3^ hybridization in the remaining bulk layer was preserved due to the constraint applied by the reforming surface layer. The foregoing process is revealed by the *sp*^3^ depth profiles of the oxidized *a*-C film shown in Fig. 4b.

**Figure 5:**
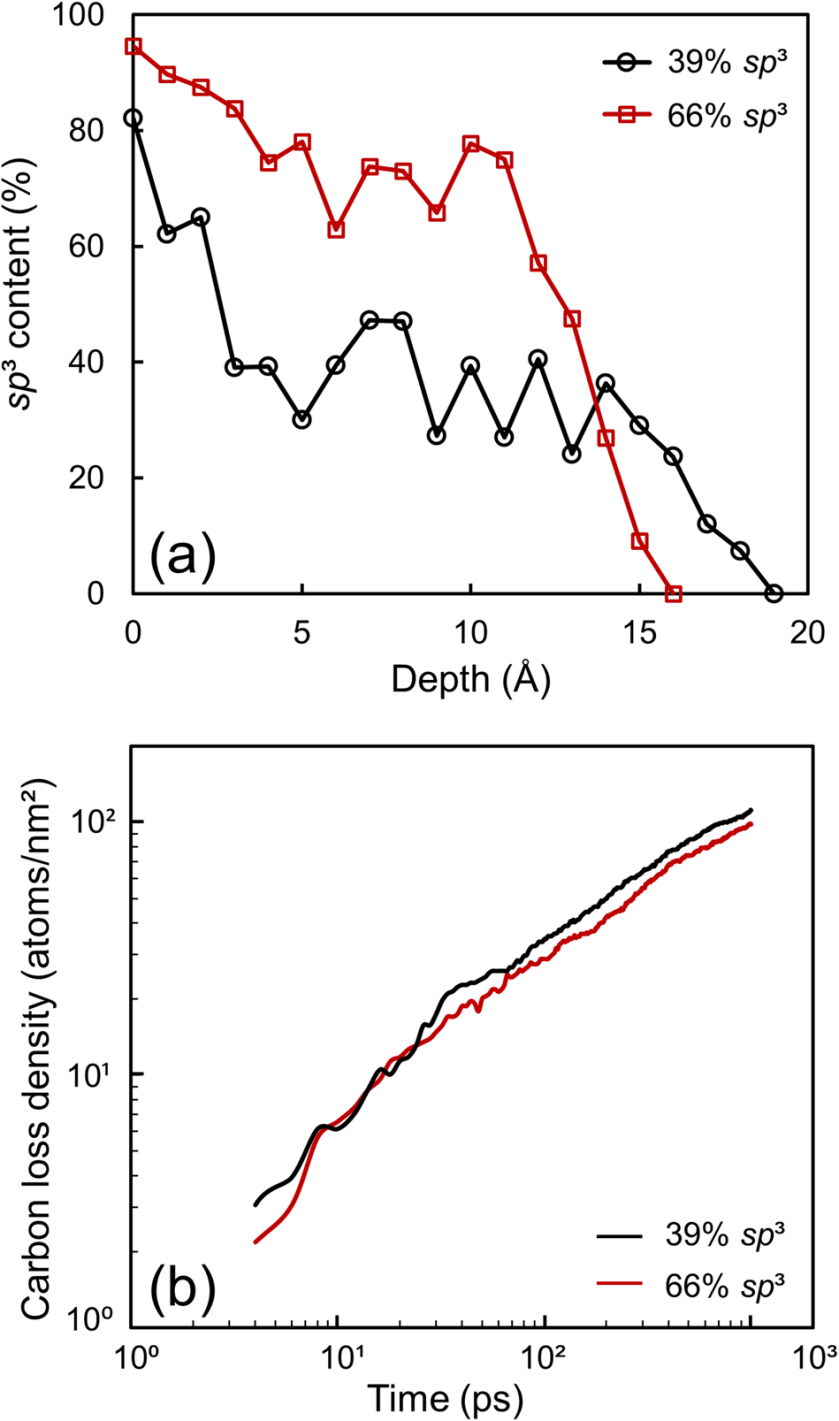
(**a**) Depth profiles of the *sp*^3^ content and (**b**) carbon atom loss density of *a*-C films with 39% and 66% *sp*^3^ contents for an oxygen kinetic energy equal to 20 eV. The zero depth position in (**a**) corresponds to the bottom atomic plane of the film (the fixed red atoms in Fig. 1).

It is widely accepted that *a*-C films with high *sp*^3^ contents demonstrate a better protection performance because they exhibit improved mechanical properties. To examine the dependence of the oxidation resistance of the film on its structure, simulations were performed with ultrathin *a*-C films with 39% and 66% *sp*^3^ contents for an oxygen kinetic energy fixed at 20 eV. Henceforth, these two films will be referred to as the low and high *sp*^3^ films, respectively, for simplicity. The *sp*^3^ depth profiles of the foregoing films obtained after oxidation, shown in Fig. 5a, illustrate the formation of surface layers with a similar thickness characterized by a sharply decreasing *sp*^3^ content towards the film surface; however, the *sp*^3^ content of the bulk layer of the high *sp*^3^ film is almost two times higher than that of the bulk layer of the low *sp*^3^ film. Interestingly, the curves of the carbon loss density versus time, shown in Fig. 5b, reveal that the rates of carbon loss of the two films are almost identical despite of the vastly different overall and bulk layer *sp*^3^ contents. These results suggest that the oxidation of ultrathin *a*-C films is confined within the surface layer. Since the formation of a surface layer with a low *sp*^3^ content is inevitable due to the previously mentioned effects of surface relaxation and less intense bombardment by energetic atoms during film growth, the results show that increasing the overall or bulk layer *sp*^3^ contents will not necessarily enhance the oxidation resistance of the film.

### Adhesion and friction characteristics and the tribo-oxidation process

Significant discrepancies in the friction behavior of *a*-C films are often encountered due to differences in the structure and operation conditions. For instance, the coefficient of friction of various types of *a*-C films has been reported to vary in the wide range of 0.003–0.7^44^. In particular, it is well established that the structure, testing method, and environmental conditions can strongly affect the tribochemical behavior of *a*-C films. The combined effects of the aforementioned factors further perplex the tribochemical behavior when the film thickness is only a few nanometers and structure changes and/or chemical reactions occur rapidly, making experimental tracking in real time a formidable task. The MD simulation results of normal and sliding contact presented in this section provide insight into the spatiotemporal changes of the friction, nanostructure, and oxidation behavior of ultrathin *a*-C films in vacuum and oxygen atmosphere.

**Figure 6:**
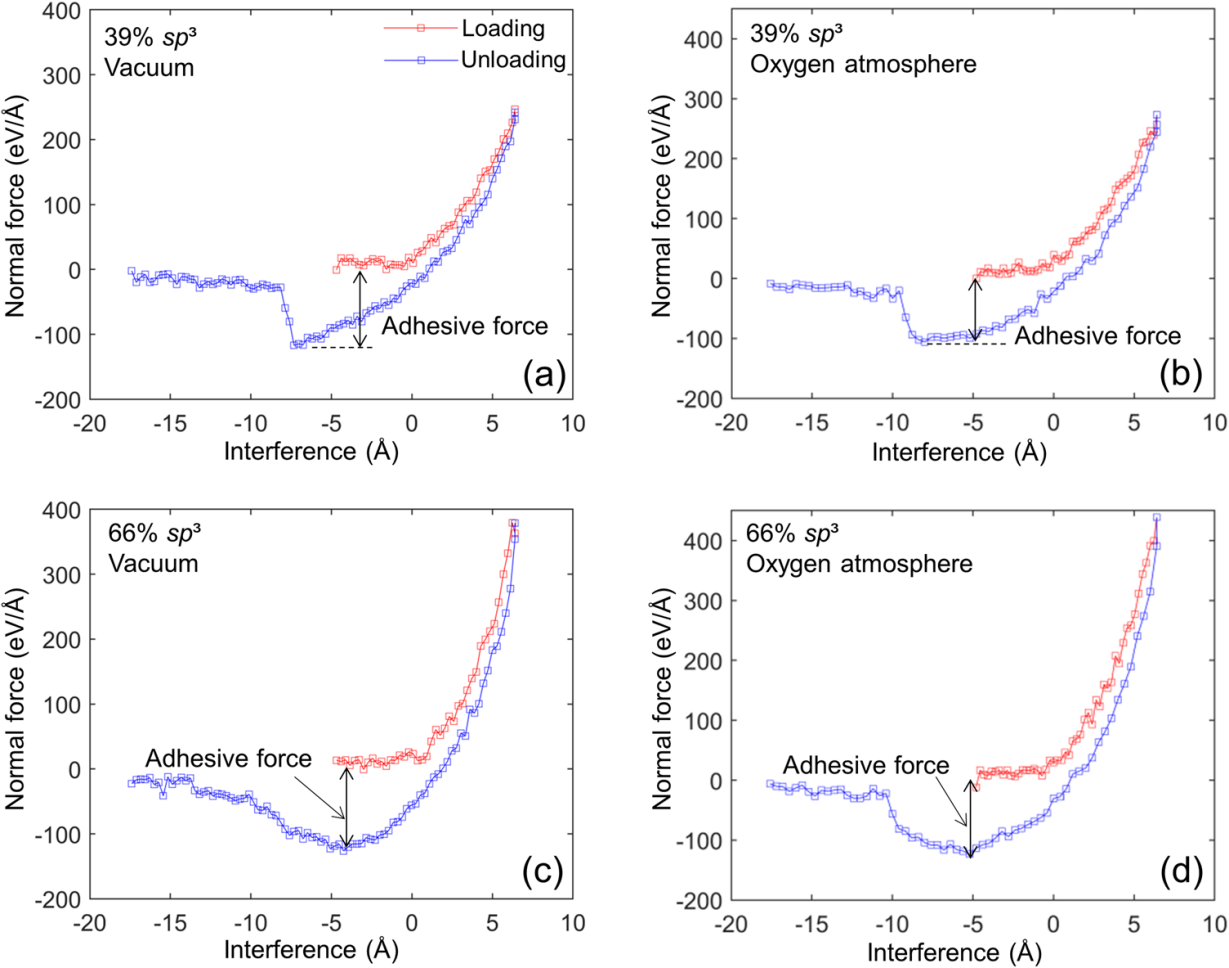
Normal force versus surface interference for two identical *a*-C films with (**a**,**b**) 39% *sp*^3^ and (**c**,**d**) 66% *sp*^3^ content interacting in vacuum and oxygen atmosphere. The negative force values represent the attractive forces generated during the retraction phase of the simulation. The maximum attractive force is referred to as the adhesive force (1 eV/Å = 1.6 nN).

Figure 6 shows the variation of the normal force with the surface interference for two similar *a*-C films having either low or high *sp*^3^ contents brought into normal contact in vacuum and oxygen atmosphere. The results reveal significant differences and also similarities of film interaction that cannot be easily captured experimentally. The hysteresis area between the loading (red) and unloading (blue) force paths indicates the accumulation of irreversible deformation, implying changes in the film nanostructure. The maximum positive (repulsive) normal force of the high *sp*^3^ films is greater than that of the low *sp*^3^ films in both vacuum and oxygen atmosphere simulation cases. This difference is attributed to the increase of both the film stiffness and deformation resistance with increasing *sp*^3^ content. The significant negative (attractive) normal forces encountered during unloading in the subzero surface interference range reveal the existence of strong attractive forces between the withdrawing films, attributed to their high affinity to bond to each other during loading. Henceforth, the maximum attractive force at which surface separation is instigated will be referred to as the adhesive force. Interfacial attractive forces may result from several types of bonding, including covalent bonds between free or dangling bonds at the contact interface of the films. This type of attractive force has been observed in the nanoindentation of *a*-C films by a diamond tip^45^. Animations of the four indentation simulation cases shown in Fig. 6 can be seen in videos A–D of the Supplementary Information (SI). These video recordings reveal that film separation during unloading is a continuous process characterized by the gradual breakage of bonds at the contact interface. The attractive force drops rapidly to zero upon the full detachment of the low *sp*^3^ films (Fig. 6a,b) because of the relatively abrupt instigation of bond breaking, as shown in videos A and B of the SI, whereas for the high *sp*^3^ films the attractive force decreases rather gradually (Fig. 6c,d) due to the progressive breakage of the bonds, as shown in videos C and D of the SI. The latter trend of the attractive force may be attributed to the more extensive destruction of the surface layer of the high *sp*^3^ films caused by more carbon atom transfer during the retraction stage, as evidenced in videos C and D of the SI. In contrast to the vastly different maximum repulsive forces, the adhesive forces are almost identical in all four simulation cases, suggesting high film reactivity due to a large amount of unsaturated carbon atoms at the film surfaces, even in the oxygen atmosphere. These unsaturated carbon atoms can easily form new bonds across the film contact interface during loading, regardless of the overall *sp*^3^ film content. This is because both low and high *sp*^3^ films possess similar *sp*^2^-dominted surface layers (Fig. 5a). It is further noted that the adhesion force of the high *sp*^3^ films was encountered sooner during unloading compared to the low *sp*^3^ films, presumably due to the greater elastic strain energy release of the stiffer films with high *sp*^3^ content. Another interesting observation is that oxygen adsorption does not appear to have an effect on the magnitude of the adhesive force. Different from hydrogen and water vapor, oxygen shows very diverse effects on the surface passivation of *a*-C films^46^. The dissociative oxygen adsorption due to the elevated temperature in the oxygen atmosphere simulations resulted in oxygen adsorption at the film surface in the form of atomic oxygen. At equilibrium, the adsorbed oxygen atoms were fewer than the surface carbon atoms due to the high temperature of the films, resulting in partial surface passivation. Thus, because of the dependence of the adhesive force on partial surface coverage and the larger van der Waals diameter of the oxygen atom than the carbon atom bond length, a similar adhesive force was encountered in vacuum and oxygen atmosphere^46,47^.

**Figure 7:**
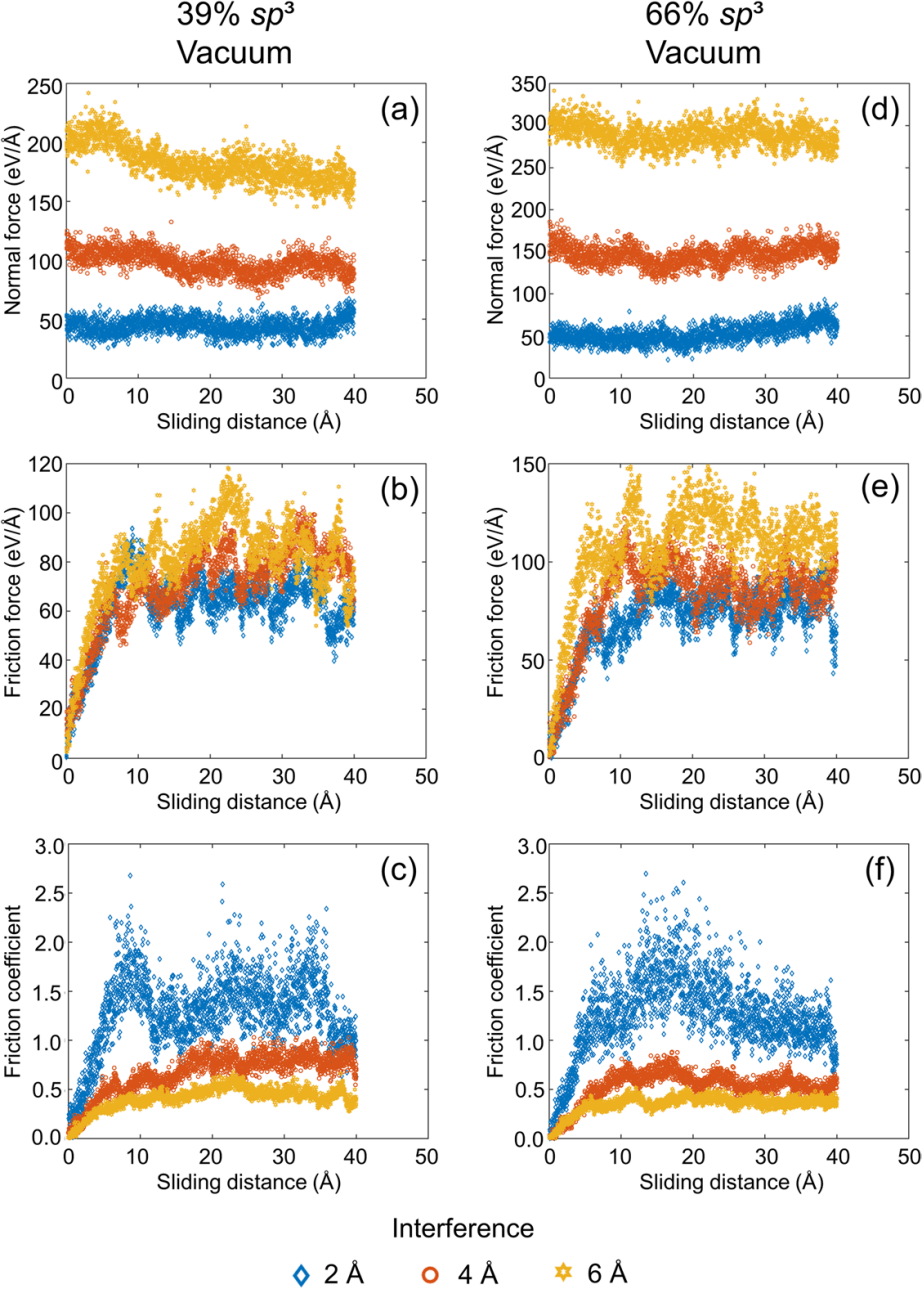
Normal force, friction force, and coefficient of friction versus sliding distance and surface interference for two identical *a*-C films with (**a**–**c**) 39% *sp*^3^ and (**d**–**f**) 66% *sp*^3^ contents sliding against each other in vacuum (1 eV/Å = 1.6 nN).

**Figure 8:**
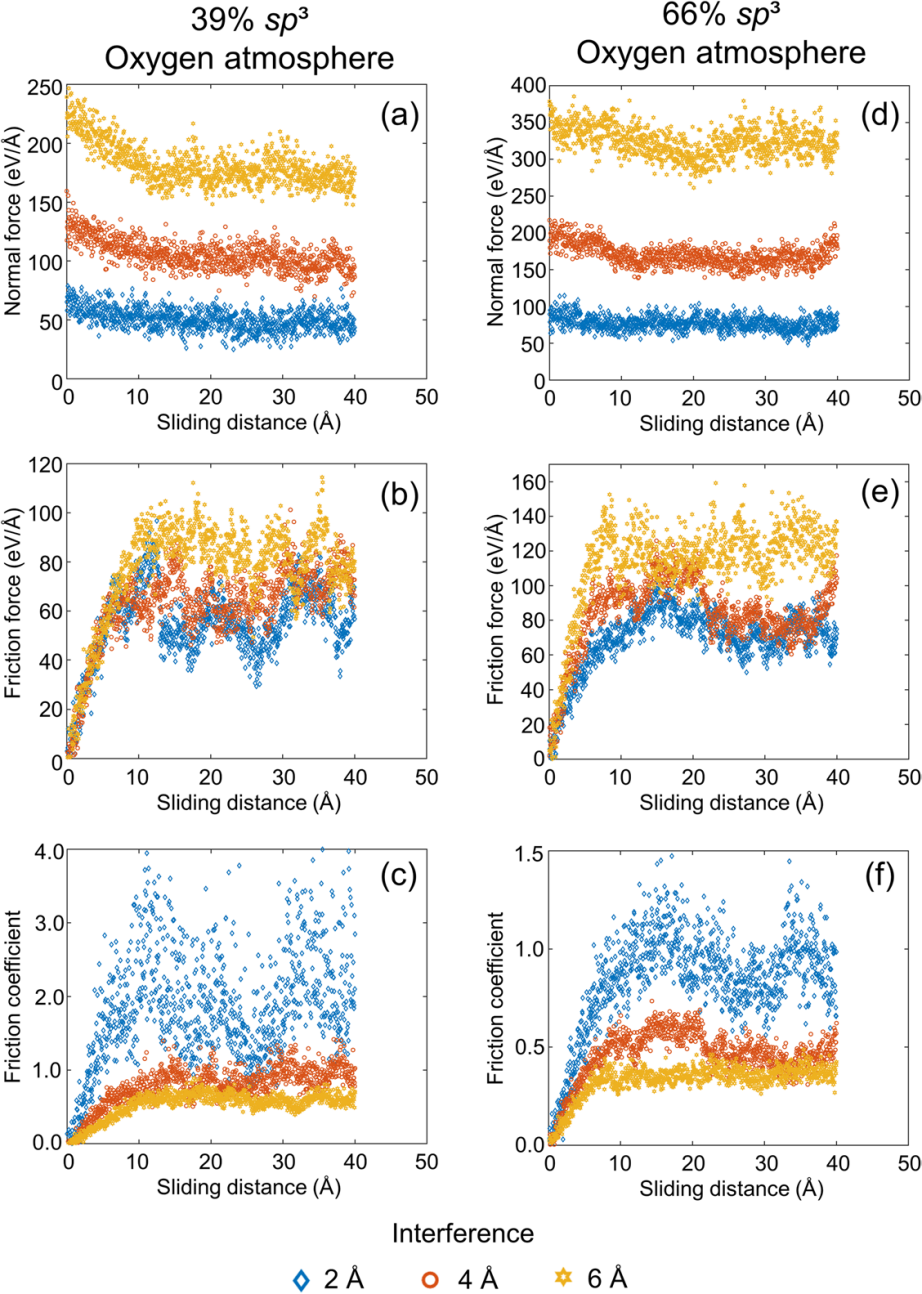
Normal force, friction force, and coefficient of friction versus sliding distance and surface interference for two identical *a*-C films with (**a**–**c**) 39% *sp*^3^ and (**d**–**f**) 66% *sp*^3^ contents sliding against each other in oxygen atmosphere (1 eV/Å = 1.6 nN).

The significantly more dynamic sliding process than normal contact makes experimental tracking of changes in the structure and tribological properties of ultrathin films even more challenging. The variations of the normal force, friction force, and friction coefficient with sliding distance in vacuum and oxygen atmosphere, shown in Figs. 7 and 8, respectively, provide insight into the contact behavior and the evolution of friction during sliding. It is noted that the results of the sliding simulations were not biased by frictional heating because the temperature in each film was maintained at 573 K by the Berendsen thermostats. In addition, the animations of the sliding simulation cases for low and high *sp*^3^ contents, 6 Å interference, and vacuum or oxygen atmosphere, shown in videos E–H of the SI, reveal atom intermixing and continuous formation and breakage of bonds at the contact interface. As expected, the normal force increased with the surface interference in all simulations (Figs. 7a,d and 8a,d). Alternatively, the friction force showed a two-stage response in all cases, that is, an initial transient stage where the friction force increased rapidly up to a sliding distance of ∼10 Å and a steady-state stage characterized by significant force fluctuations (Figs. 7b,e and 8b,e). The intense surface atom interactions occurring during the transient stage of sliding due to the highly reactive surfaces led to the repetitive formation and breakage of bonds and the rearrangement of surface atoms. In fact, the film contact interface was indistinguishable at the end of the transient stage of sliding due to the intermixing of the atoms of the surface layers of the sliding films, as evidenced in videos E–H of the SI. The rearrangement of the surface atoms also explains the decrease of the normal force with increasing sliding distance for the low *sp*^3^ films and large interference (i.e., 4 and 6 Å in Figs. 7a and 8a). During sliding, the atoms in the less dense film surface migrated to lower energy sites, relaxing the normal force in the low *sp*^3^ films at large interference. A relaxation of the normal force of the high *sp*^3^ films (Figs. 7d and 8d) did not occur due to the considerably less free space for the rearrangement of surface atoms produced by the more intense compressive stress field generated in the stiffer nanostructures of these films.

Shear-induced breakage of interface bonds occurred during sliding as the shear surface traction exceeded the attractive forces. Thus, progressively more intense fluctuations of the friction force were instigated with increasing interference as new bonds were disrupted and formed during sliding. Due to the more pronounced fluctuations of the friction force compared to the normal force, the friction coefficient exhibited even more intense fluctuations after the initial transient stage of the friction force. The decrease of the average steady-state friction coefficient with increasing surface interference (normal force), shown in Figs. 7c,f and 8c,f, reveals a nonlinear relation between the normal and friction forces. A nonlinear friction-normal force relation has also been observed in a previous study^48^ and is indicative of the breakdown of Amontons’ first law of friction at the atomic level. Moreover, a small variation of the friction force due to sliding of an active *a*-C film surface against a diamond surface has been reported for a wide range of the normal force^47^.

**Figure 9:**
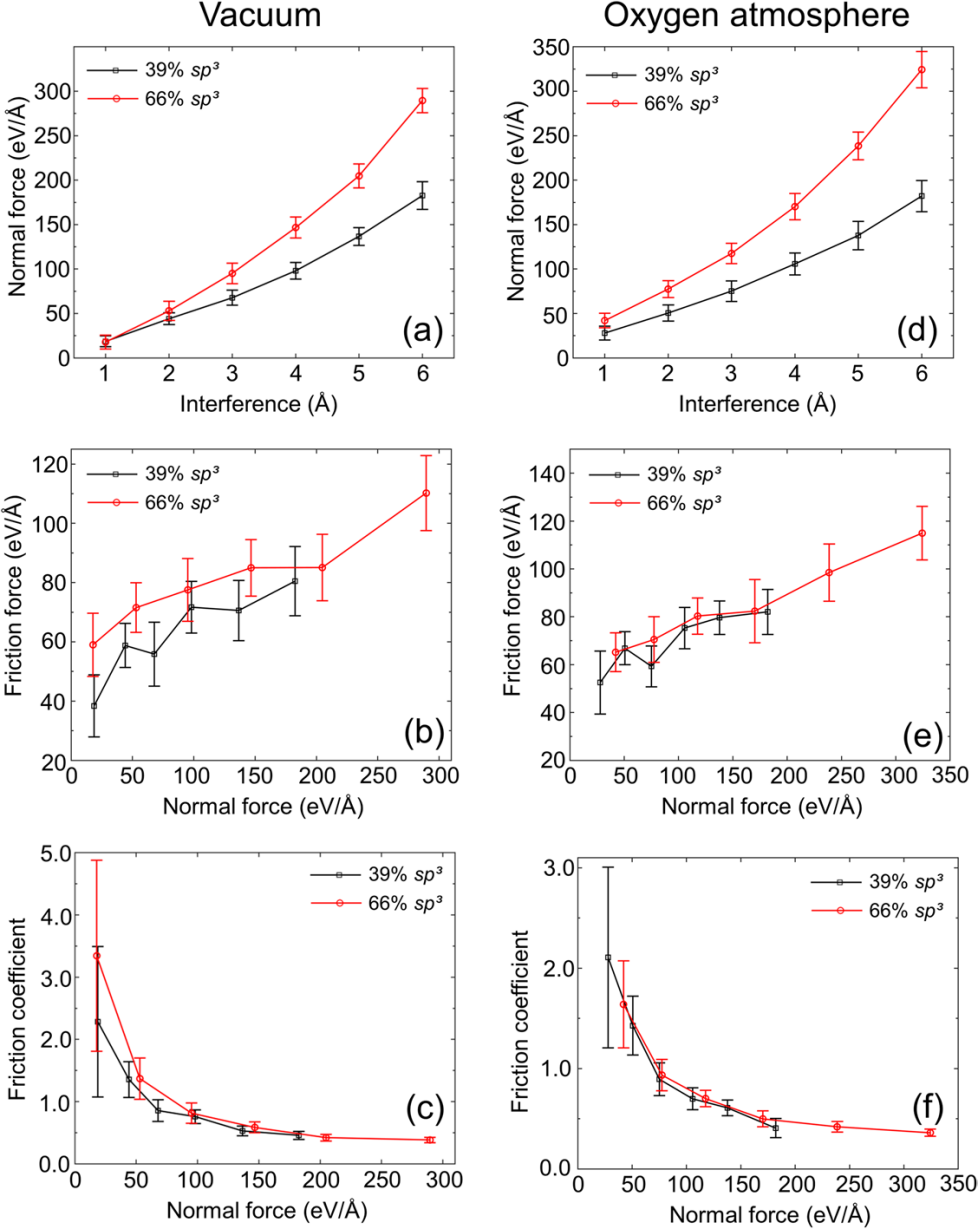
(**a**,**d**) Steady-state normal force versus surface interference, and (**b**,**e**) friction force and (**c**,**d**) coefficient of friction versus normal force for two identical *a*-C films with 39% and 66% *sp*^3^ contents sliding against each other in vacuum and oxygen atmosphere (1 eV/Å = 1.6 nN).

The simulation results shown in Fig. 9 illuminate the effects of the environment and *sp*^3^ fraction on the dependence of the normal force on the surface interference and the friction behavior on the normal force. The monotonic increase of the normal force with the surface interference (Fig. 9a,d) is attributed to a hardening effect caused by the densification of the compressed films and the greater contribution of the stronger bulk layer to the penetration resistance that became more pronounced with increasing surface interference. The higher normal force of the high *sp*^3^ films than the low *sp*^3^ films at a given interference is attributed to the proliferation of the deformation resistance with *sp*^3^ hybridization, in agreement with the loading paths of the normal force shown in Fig. 6. The higher normal and friction forces in oxygen atmosphere than those in vacuum are ascribed to the force contributed by the adsorbed oxygen atoms. As mentioned earlier, the friction force exhibited more significant variations during sliding compared to the normal force (Figs. 7 and 8). The nonlinear relation between the normal and friction forces reflected by the nonlinear variation of the friction coefficient with the normal force (Fig. 9c,f) indicates a deviation of atomic-scale friction from the classical friction theory of Amontons according to which the friction force generated between two sliding macroscopic bodies is linearly proportional to the normal force due to the constancy of the friction coefficient^49^. However, power-law (Hertz theory) or sublinear (Maugis-Dugdale model) relations between friction and normal forces^50^ have been presented in adhesion-based friction theories. The results shown in Fig. 9b,e for low and high *sp*^3^ films sliding in vacuum and oxygen atmosphere, respectively, reveal a sublinear relation between normal and friction forces for a normal force less than 200 eV/Å, in agreement with the foregoing adhesion-based friction theory^50^. The existence of a sublinear force relation is also supported by the nonlinear decrease of the friction coefficient with normal force (Fig. 9c,f). The very high friction coefficients obtained for a low normal force are illustrative of the differences between macroscopic and atomic-scale friction, where long-range attractive forces contribute significantly to the magnitude of the applied normal force. The lower steady-state friction coefficients obtained in the low range of the normal force for sliding in oxygen atmosphere than vacuum are attributed to the adsorbed oxygen atoms, which reduced the number of the stronger carbon–carbon bonds that readily formed in vacuum.

**Figure 10:**
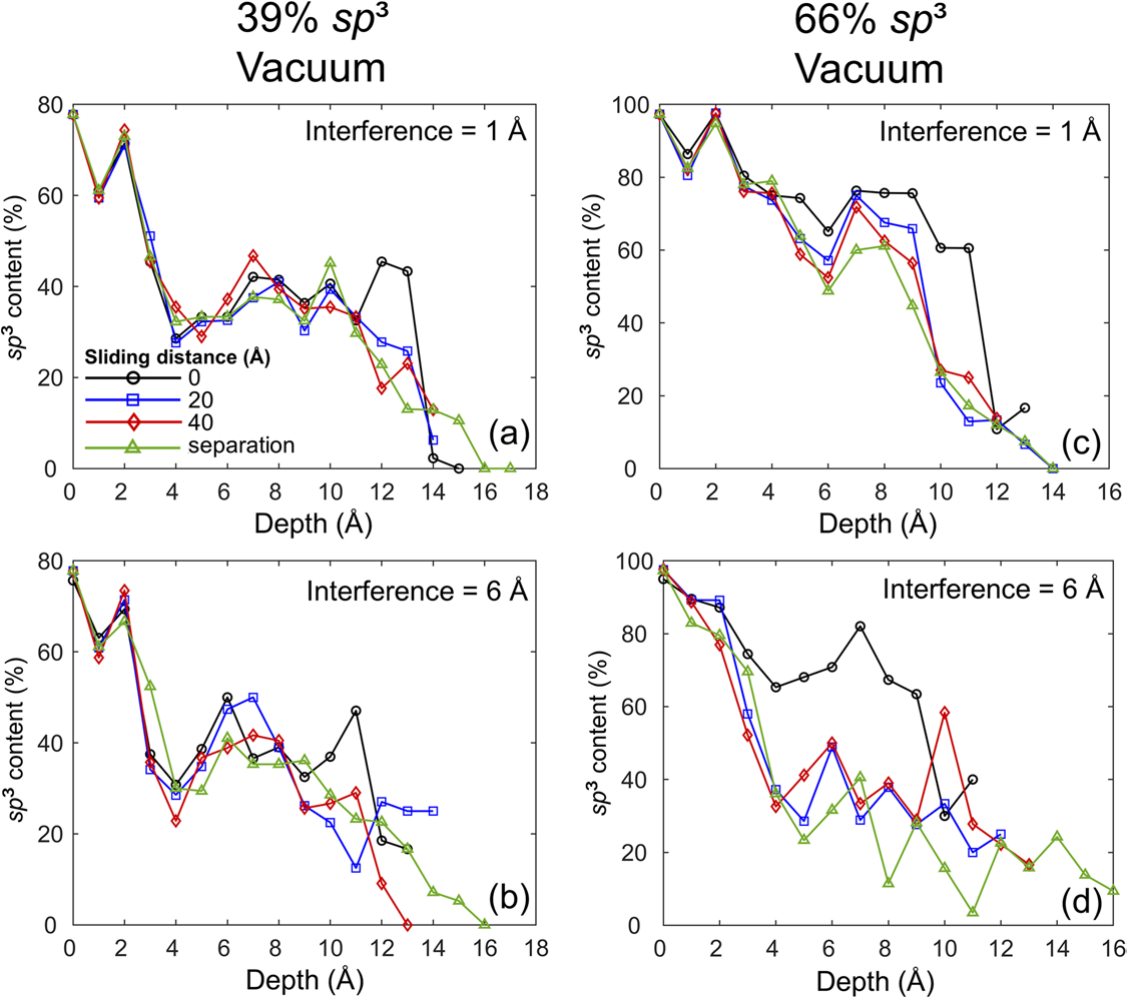
Depth profiles of the *sp*^3^ content of two identical *a*-C films with 39% and 66% *sp*^3^ contents sliding against each other in vacuum for a surface interference equal to 1 and 6 Å obtained after a sliding distance equal to 0 (pure normal contact), 20, and 40 Å and after film separation.

A significant oxidation effect on the nanostructure (hybridization state) of the *a*-C films was found under oxidizing conditions that did not involve surface contact (Fig. 4b). The shear traction generated by the sliding process may produce even more pronounced structural changes, especially for a large surface interference (high normal force). To investigate the progression of structural changes through the film thickness due to sliding, the *sp*^3^ depth profiles of both low and high *sp*^3^ films were obtained at different stages of sliding in vacuum (to avoid complication from adsorbed oxygen atoms) and also after film separation for a surface interference of 1 and 6 Å, and the results are compared in Fig. 10. A significant difference between the *sp*^3^ depth profiles of non-oxidized *a*-C films obtained before film contact (Fig. 4b) with those of similar films in normal contact, i.e., zero sliding distance (Fig. 10c,d), is that the latter films exhibited nonzero *sp*^3^ fractions at their surfaces, indicating *sp*^3^ hybridization of unsaturated surface carbon atoms under the compressive mechanical environment produced by the normal force. Sliding caused the formation and breakage of carbon atom bonds and the rearrangement and intermixing of carbon atoms in the proximity of the sliding contact interface (see videos E–H of the SI). The intense atomic interactions affected not only the near-surface region of the films but also regions remote from the contact interface, especially for relatively large surface interference (high normal force). Indeed, a noticeable drop in *sp*^3^ hybridization occurred in the depth range of 4–12 Å with increasing sliding distance and, more significantly, after the separation of the films for a surface interference equal to 1 Å (Fig. 10c). A more dramatic decrease of the *sp*^3^ fraction in the depth range of 4–9 Å occurred with the increase of the surface interference to 6 Å (Fig. 10d) and after sliding for a distance of 20 Å. The significant structural changes remote from the contact interface reveal shear-induced *sp*^3^-to-*sp*^2^ rehybridization in the bulk layer of the high *sp*^3^ films (Fig. 10c,d). However, structural changes in the bulk layer of the low *sp*^3^ films were minimal for both small and large surface interferences (Fig. 10a,b), presumably because *sp*^2^ hybridization is energetically more stable than *sp*^3^ hybridization. This is also evidenced by the similar *sp*^3^ depth profiles of the high and low *sp*^3^ films obtained after sliding for 40 Å (Fig. 10b and d, respectively). After film separation, all of the *sp*^3^ depth profiles showed an increase in film thickness due to irreversible film stretching during the detachment of the films. Despite sliding-induced *sp*^3^-to-*sp*^2^ rehybridization, the friction did not decrease because of the highly reactive film surfaces produced by the continuous breakage of surface bonds and the relatively short sliding distance for activating extensive shear-induced surface graphitization^51^.

**Figure 11:**
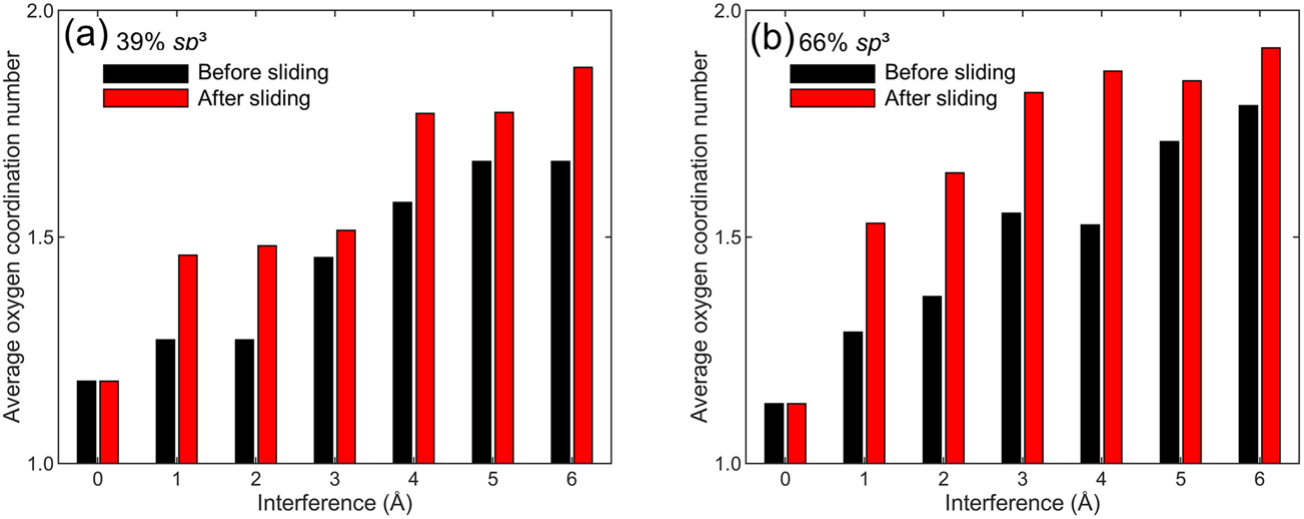
Average coordination number of oxygen atoms adsorbed into the surfaces of two identical *a*-C films with 39% and 66% *sp*^3^ contents versus surface interference. The increase of the coordination number with surface interference indicates an increase of oxygen-carbon bonds, implying an enhancement of film oxidation with intensifying film interaction instigated by the sliding process.

Although oxygen surface adsorption did not produce a first-order effect on friction because of the partial film surface coverage (passivation), shear-induced chemical reactivity resulted in tribo-oxidation in the sliding simulations. This was demonstrated by the changes in the average oxygen coordination number of adsorbed oxygen atoms, calculated by averaging the numbers of the nearest neighboring carbon atoms of all adsorbed oxygen atoms. Figure 11 shows the average oxygen coordination number before and after sliding of low and high *sp*^3^ films for a surface interference in the range of 0–6 Å. The increase of the average oxygen coordination number with surface interference indicates that film interpenetration during normal contact enhanced carbon–oxygen bond formation and, in turn, the oxidation of both low and high *sp*^3^ films. The additional significant increase of the average oxygen coordination number caused by sliding illuminates the important role of shear traction in tribo-oxidation. For low and high *sp*^3^ films sliding at a surface interference of 6 Å, the average oxygen coordination number is close to 2, implying chemical reaction of almost all oxygen atoms. These results confirm that tribo-oxidation reactions activated by persistent sliding in oxidizing environments can greatly modify the nanostructure and tribological properties of *a*-C films.

## Conclusions

MD simulations were performed to elucidate the effects of nanostructure (hybridization state), surface interference (normal force), and oxygen adsorption (surface reactivity) on the oxidation behavior and adhesion and friction characteristics of ultrathin *a*-C films. The MD analysis of film oxidation by energetic oxygen molecules revealed the existence of a critical oxygen kinetic energy below which carbon loss did not occur and that steady-state oxygen adsorption was independent of the oxygen kinetic energy due to the saturation of the film surfaces by oxygen. Film oxidation comprised the removal of carbon atoms from the low *sp*^3^ surface layer of the films as gaseous species. The relaxation of the underlying *sp*^3^-rich bulk layer, which was propelled by the gradual depletion of the surface layer by the oxidation process, activated *sp*^3^-to-*sp*^2^ hybridization in the topmost region of the bulk layer that replenished the surface layer. This oxidation mechanism perpetuated itself, resulting in an exponential relation of the carbon loss rate. These simulations confirmed that oxidation of ultrathin *a*-C films possessing a layered structure comprised of an *sp*^3^-rich bulk layer and an *sp*^2^-rich surface layer is a surface-dominated process controlled by the oxidation and removal of the surface layer and its progressive reformation via a localized phase transformation process involving *sp*^3^-to-*sp*^2^ rehybridization instigated in the bulk layer adjacent to the interface with the surface layer.

The MD results of normal contact between identical *a*-C films revealed similar adhesive forces in vacuum and oxygen atmosphere due to the partial passivation of the contacting film surfaces by oxygen. Simulations of *a*-C films with similar nanostructures (hybridizations) sliding against each other in vacuum or oxygen atmosphere demonstrated intensifying normal and friction forces and decreasing friction coefficient with increasing surface interference, revealing a departure from Amontons’ first friction law at the atomic scale. Atomic rearrangement and intermixing in conjunction with shear-induced *sp*^3^-to-*sp*^2^ rehybridization occurred during sliding, especially for high *sp*^3^ *a*-C films. Relatively higher friction coefficient values were obtained for sliding in vacuum than oxygen atmosphere due to the formation of more carbon–carbon bonds in vacuum compared to the partially oxygen-passivated film surfaces in oxygen atmosphere. Sliding (shear) traction enhanced film oxidation (tribo-oxidation) even in an oxygen lean environment. From a fundamental tribo-chemistry perspective, the present study provides a computational framework for investigating other thin-film systems exposed to various oxidizing and contact conditions and useful guidance for the design of tribo-oxidation experiments.

## Supplementary Information

Supplementary Video A.

Supplementary Video B.

Supplementary Video C.

Supplementary Video D.

Supplementary Video E.

Supplementary Video F.

Supplementary Video G.

Supplementary Video H.

Supplementary information.

## Data Availability

The datasets generated during and/or analyzed during the current study are available from the corresponding author on reasonable request.
