# Supplementary material for: A molecular dynamics study of the oxidation mechanism, nanostructure evolution, and friction characteristics of ultrathin amorphous carbon films in vacuum and oxygen atmosphere
Source: Sci Rep. 2021 Feb 16;11:3914. doi: 10.1038/s41598-021-81659-w (PMC7886871; doi:10.1038/s41598-021-81659-w)
Supplement: Supplementary file 9 — Supplementary information. [file 41598_2021_81659_MOESM9_ESM.docx]

**A molecular dynamics study of the oxidation mechanism, nanostructure evolution, and friction characteristics of ultrathin amorphous carbon films in vacuum and oxygen atmosphere**

Shengxi Wang & Kyriakos Komvopoulos

Department of Mechanical Engineering, University of California, Berkeley, CA 94720, USA

**Supplementary Information**

The following videos of normal contact (A–D) and sliding contact (E–H) MD simulations of low-*sp*^3^ (39%) and high-*sp*^3^ (66%) *a*-C films interacting in vacuum and oxygen atmosphere comprise the SI of this paper.

**Video A.** Normal contact of low *sp*^3^ *a*-C films in vacuum.

**Video B.** Normal contact of low *sp*^3^ *a*-C films in oxygen atmosphere.

**Video C.** Normal contact of high *sp*^3^ *a*-C films in vacuum.

**Video D.** Normal contact of high *sp*^3^ *a*-C films in oxygen atmosphere.

**Video E.** Sliding contact of low *sp*^3^ *a*-C films in vacuum (surface interference = 6 Å).

**Video F.** Sliding contact of high *sp*^3^ *a*-C films in vacuum (surface interference = 6 Å).

**Video G.** Sliding contact of low *sp*^3^ a-C films in oxygen atmosphere (surface interference = 6 Å).

**Video H.** Sliding contact of high *sp*^3^ *a*-C films in oxygen atmosphere (surface interference = 6Å).
